# Supplementary material for: IL‐21 has a critical role in establishing germinal centers by amplifying early B cell proliferation
Source: EMBO Rep. 2022 Jul 8;23(9):e54677. doi: 10.15252/embr.202254677 (PMC9442303; doi:10.15252/embr.202254677)
Supplement: Supplementary file 1 — Expanded View Figures PDF [file EMBR-23-e54677-s002.pdf]

# Expanded View Figures

**Figure EV1. $SW_{HEL}$ B cells characterization and flow cytometry gating strategy for Figs 1–3.**

A–C Representative analysis of splenic lymphocytes from a $SW_{HEL}$ mouse by flow cytometry showing $HEL^{2X}OVA_{pep}$ binding (A), eGFP expression (B) and HEL-$OVA_{pep}$ variant binding to $SW_{HEL}$ B cells (C).

D Exemplary electronic gating strategy for Fig 1B–E to identify WT ($GFP^{-}$) and *Il21r*$^{-/-}$ ($GFP^{+}$) $SW_{HEL}$ B cells.

E Exemplary electronic gating strategy for Figs 2B and D, and 3C. $SW_{HEL}$ B cells were identified by CTV and $HEL^{2X}OVA_{pep}$ antigen-binding. eGFP was used to distinguish WT and *Il21r* $^{-/-}$ cells. Blue gates were used for analysis in Fig 1I and red gates for Fig 3C.

 © 2022 The Authors

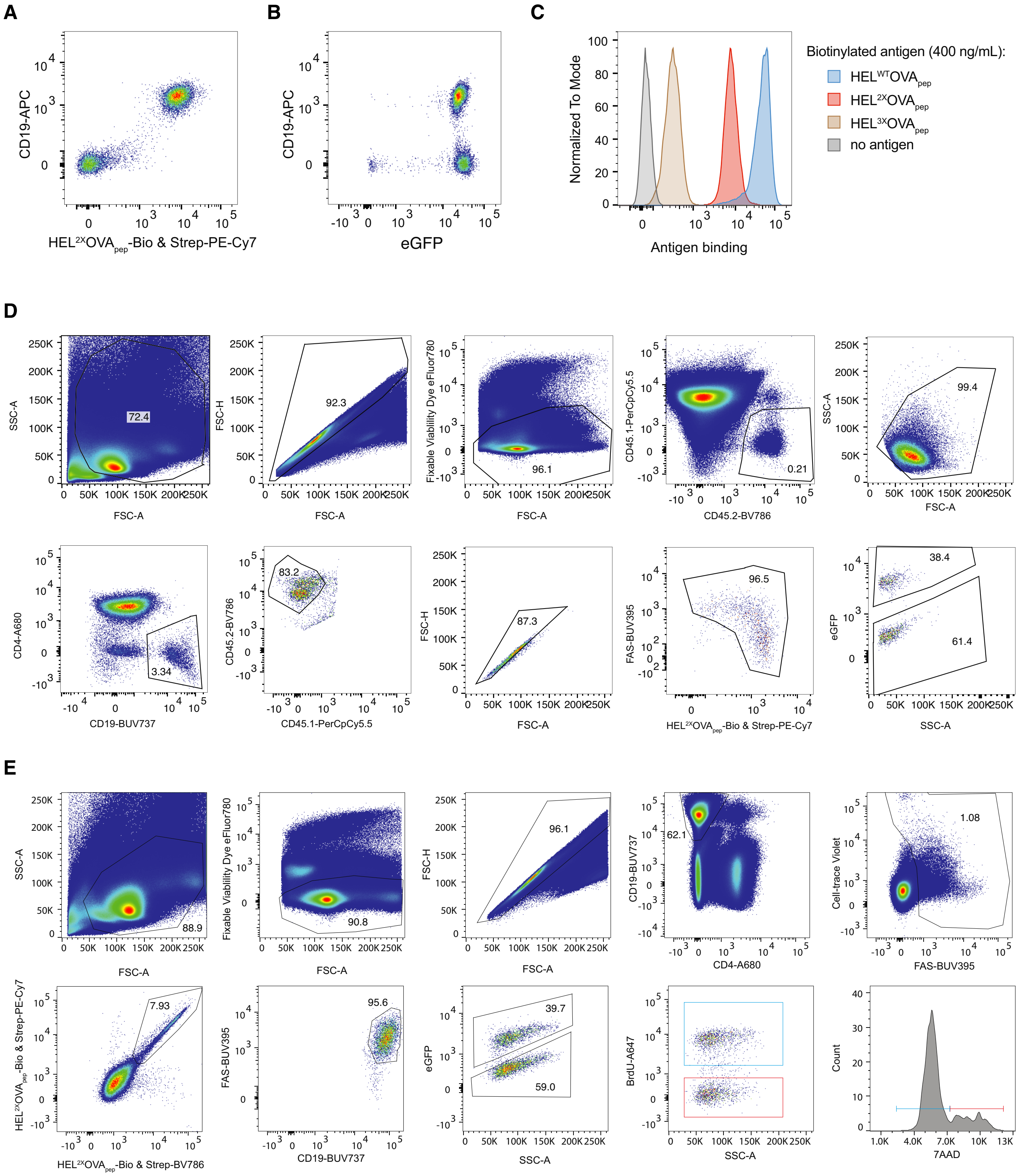
A
CD19-APC
HEL2XOVApep-Bio & Strep-PE-Cy7
B
CD19-APC
eGFP
C
Normalized To Mode
Antigen binding
Biotinylated antigen (400 ng/mL):
HELWTOVApep
HEL2XOVApep
HEL3XOVApep
no antigen
D
SSC-A
FSC-A
72.4
FSC-H
92.3
Fixable Viability Dye eFluor780
96.1
CD45.1-PerCpCy5.5
CD45.2-BV786
0.21
99.4
CD4-A680
CD19-BUV737
3.34
CD45.1-PerCpCy5.5
83.2
87.3
FAS-BUV395
96.5
eGFP
38.4
61.4
E
88.9
90.8
96.1
CD19-BUV737
CD4-A680
62.1
Cell-trace Violet
FAS-BUV395
1.08
HEL2XOVApep-Bio & Strep-PE-Cy7
HEL2XOVApep-Bio & Strep-BV786
7.93
95.6
39.7
59.0
BrdU-A647
Count
7AAD

**Figure EV1.**

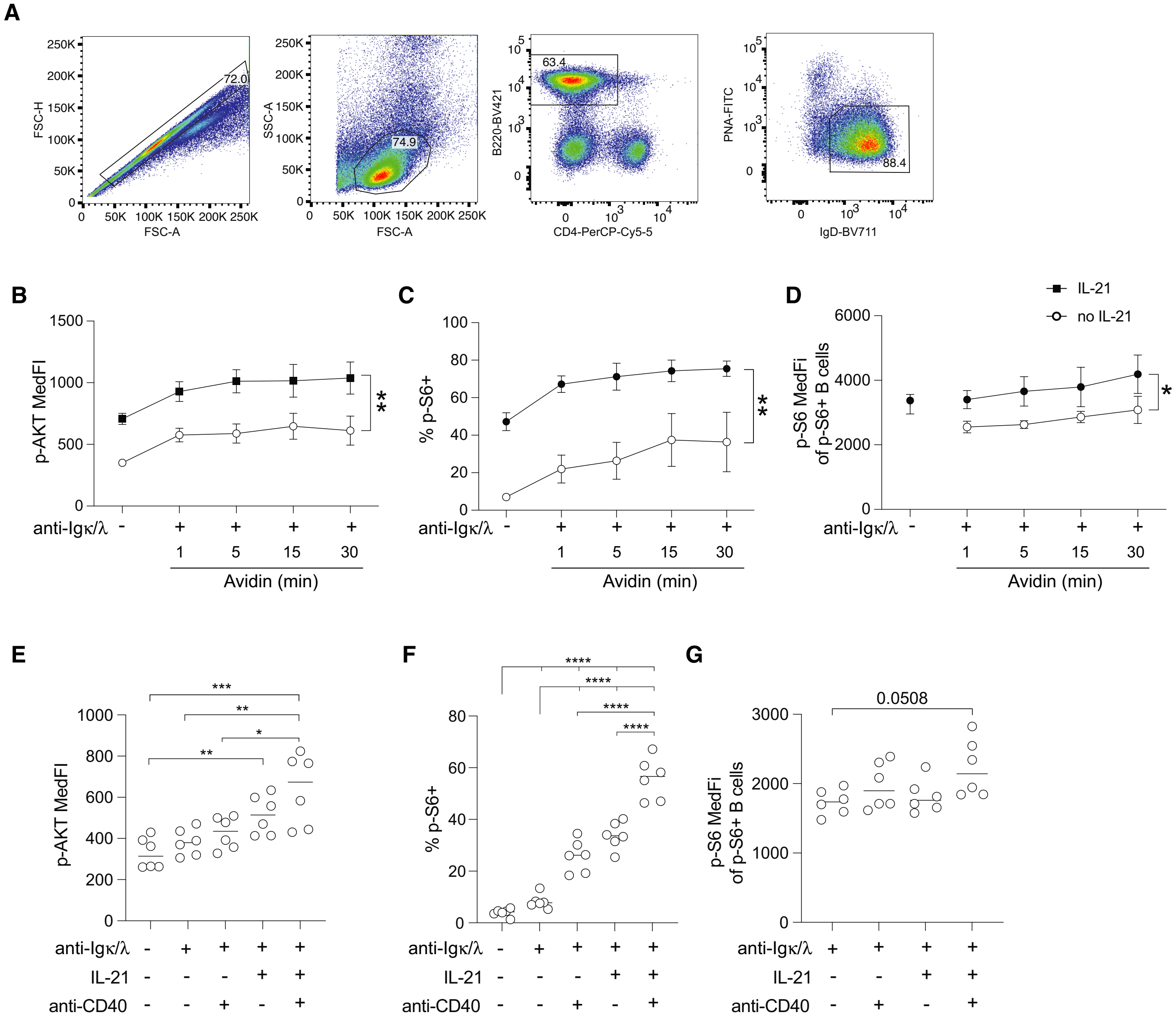


**Figure EV2. Flow cytometry gating strategy for Fig 4 and phosphoflow analysis.**

A Exemplary electronic gating strategy as applied in Fig 4 to identify naïve B cells following *in vitro* culture and phosphoflow staining.

B–D Phosphoflow analysis of naïve WT B cells following *in vitro* culture for 3 h with or without IL-21 (20 ng/ml) and with or without biotinylated anti-Igκ + anti-Igλ for the final 20 min followed by avidin-mediated BCR cross-linking for the time indicated. Median p-AKT fluorescence intensity (B), the proportion of p-S6-positive cells (C) and (D) their median p-S6 fluorescence intensity are shown.

E–G Phosphoflow analysis of naïve WT B cells following *in vitro* culture for 20 min with or without anti-Igκ + anti-Igλ followed by 30 min avidin-mediated BCR cross-linking. IL-21 and anti-CD40 were added for the final 1.5 or 1 h, respectively. Median p-AKT fluorescence intensity (E), the proportion of p-S6-positive cells (F), and their median p-S6 fluorescence intensity (G) are shown.

Data information: Data in (B–D) are pooled from four biological replicates ($n$ = 4) showing mean ± SD. Data in (E–G) show six biological replicates ($n$ = 6). Statistical analysis by two-way ANOVA (B-D) or one-way ANOVA with Tukey's post-test (E–G). *$P \le 0.05$; **$P \le 0.01$; ***$P \le 0.001$; ****$P \le 0.0001$.

 © 2022 The Authors

**Figure EV3. Flow cytometry gating strategy for Fig 5.**

A Exemplary electronic gating strategy for Fig 5B and C. OTII T cells were identified based on their co-expression of TCR Vα2 and β5 and Tfh cells by co-expression of PD-1 and CXCR5.

B Gating strategy for Fig 5D–G. Gates 1–4 were activated in SpectroFlo software after which CD45.2-positive cells were exported for analysis using FlowJo.

C Proliferation analysis by CTV dilution. WT or *Il21r*$^{-/-}$ $SW_{HEL}$ B cells from individual mice were concatenated into one file per experiment. CTV division peaks were then identified using FlowJo's Proliferation tool by first identifying peaks 0–7 and then 8–11+. Gray bars indicate gate used to set the initial peak (0 or 4). For statistical analysis in Fig 5E, the resultant electronic gates for each CTV division were then applied to individual mice.

© 2022 The Authors

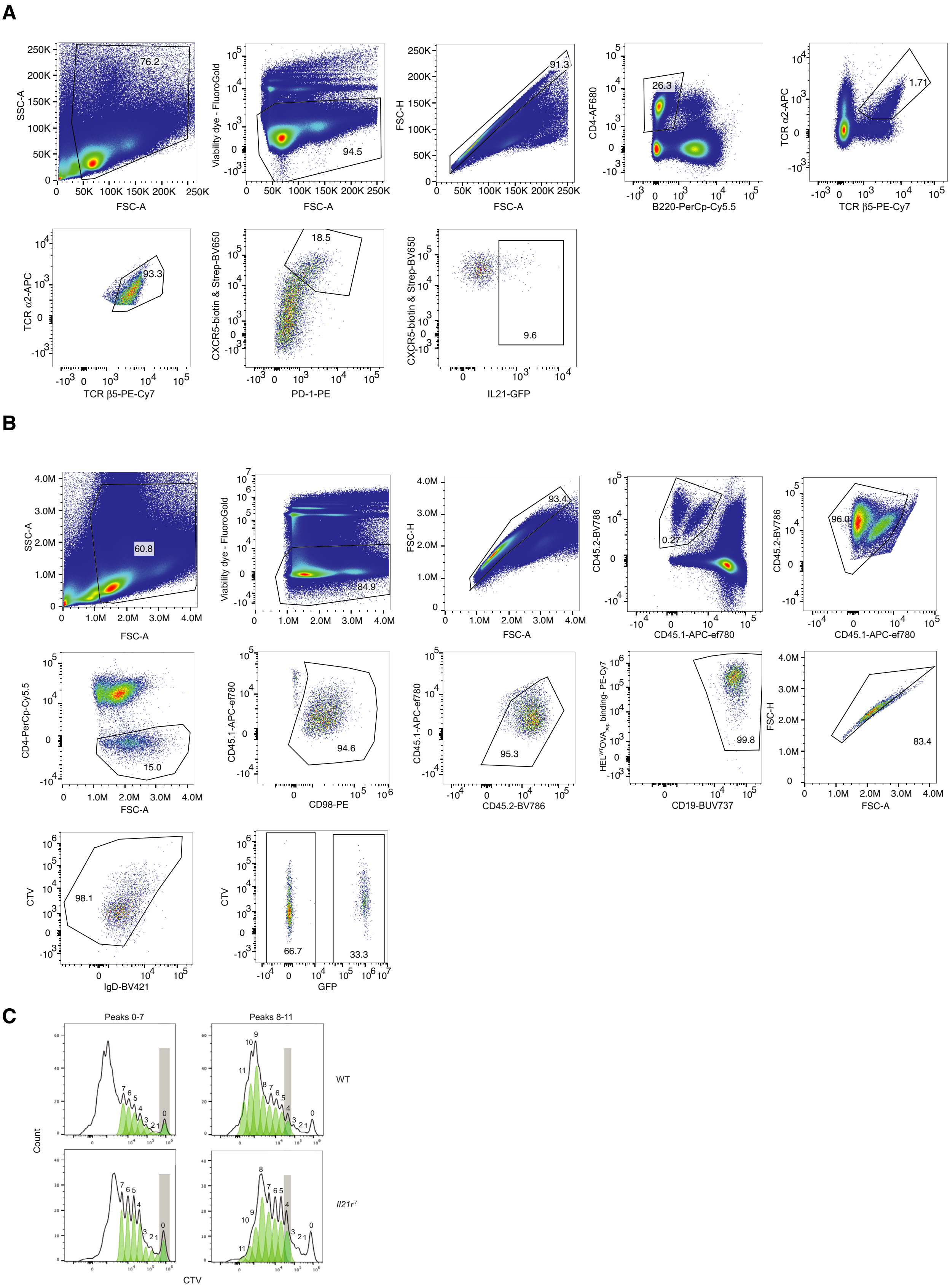


**Figure EV3.**

 © 2022 The Authors

**Figure EV4. Flow cytometry gating strategy and additional data for Fig 6.**

A Gating strategy for Fig 6. Initial gating in SpectroFlo software as shown in Fig EV3, after which CD45.2-positive cells were exported for subsequent analysis in FlowJo. Red gates indicate gates to identify plasma cells.

B–D Additional data for Fig 6 showing $SW_{HEL}$ B cells analyzed by flow cytometry. (B) Exemplary expression of FAS and GL7 on total $SW_{HEL}$ B cells. (C) Exemplary CD86 and CXCR4 expression on WT or *Il21r*$^{-/-}$ $SW_{HEL}$ GC B cells showing gates to identify LZ cells. (D) Quantification of frequency of light zone GC B cells.

Data information: Data in (D) were pooled from two independent experiments ($n$ = 6–10 biological replicates) with statistical analysis by multiple paired $t$-tests with $P$-values corrected for multiple comparisons using Holm–Šídák method. ****$P \leq 0.0001$.

© 2022 The Authors 

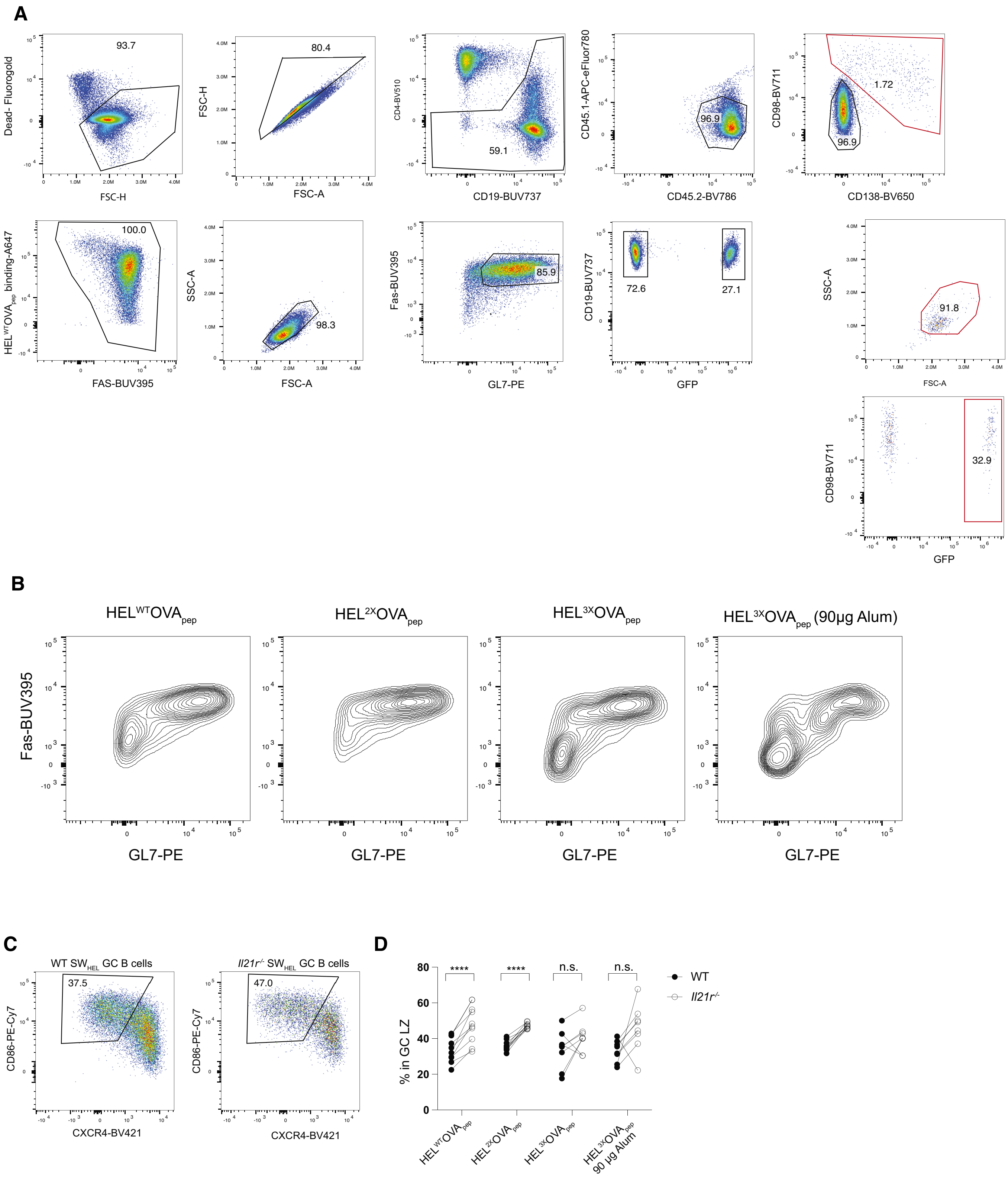


**Figure EV4.**

 © 2022 The Authors
