# Supplementary material for: IL‐21 has a critical role in establishing germinal centers by amplifying early B cell proliferation
Source: EMBO Rep. 2022 Jul 8;23(9):e54677. doi: 10.15252/embr.202254677 (PMC9442303; doi:10.15252/embr.202254677)
Supplement: Supplementary file 2 [file EMBR-23-e54677-s001.pdf]

EMBO reports

IL-21 has a critical role in establishing germinal centers by amplifying early B cell proliferation.

Alexandra Dvorscek, Craig McKenzie, Marcus Robinson, Zhoujie Ding, Catherine Pitt, Kristy O'Donnell, Dimitra Zotos, Robert Brink, David Tarlinton, and Isaak Quast
DOI: 10.15252/embr.202254677

Corresponding author(s): Isaak Quast (isaak.quast@monash.edu)

Review Timeline:

Submission Date:	17th Jan 22
Editorial Decision:	18th Jan 22
Revision Received:	9th May 22
Editorial Decision:	1st Jun 22
Revision Received:	20th Jun 22
Accepted:	22nd Jun 22

Editor: Achim Breiling

Transaction Report: This manuscript was transferred to EMBO reports following peer review at The EMBO Journal.

(Note: With the exception of the correction of typographical or spelling errors that could be a source of ambiguity, letters and reports are not edited. Depending on transfer agreements, referee reports obtained elsewhere may or may not be included in this compilation. Referee reports are anonymous unless the Referee chooses to sign their reports.)

Dear Dr. Quast,

Thank you for transferring your manuscript to EMBO reports. I now went through your manuscript, the referee reports from The EMBO Journal (attached again below) and your revision plan (point-by-point response). The referees have raised several concerns and suggestions to improve the manuscript, or to strengthen the data and the conclusions drawn.

EMBO reports emphasizes novel functional over detailed mechanistic insight, but asks for clear physiological relevance of the findings, and strong experimental support of the major conclusions. It will thus be necessary that in a revised manuscript you address all the points questioning the main conclusions of the study, and all technical concerns, or points regarding the experimental design, model systems used, or data presentation.

Given the constructive referee comments, we would like to invite you to further revise your manuscript with the understanding that all referee concerns must be addressed in the revised manuscript and in a detailed point-by-point response (as indicated in your revision plan). Acceptance of your manuscript will depend on a positive outcome of a final round of review at EMBO reports and will therefore depend on the completeness of your responses included in the next, final version of the manuscript.

When submitting your revised manuscript, please also carefully review the instructions that follow below.

PLEASE NOTE THAT upon resubmission revised manuscripts are subjected to an initial quality control prior to exposition to re-review. Upon failure in the initial quality control, the manuscripts are sent back to the authors, which may lead to delays. Frequent reasons for such a failure are the lack of the data availability section (please see below) and the presence of statistics based on n=2 (the authors are then asked to present scatter plots or provide more data points).

When submitting your revised manuscript, we will require:

1) a .docx formatted version of the final manuscript text (including legends for main figures, EV figures and tables), but without the figures included. Please make sure that changes are highlighted to be clearly visible. Figure legends should be compiled at the end of the manuscript text.

2) individual production quality figure files as .eps, .tif, .jpg (one file per figure), of main figures and EV figures. Please upload these as separate, individual files upon re-submission.

The Expanded View format, which will be displayed in the main HTML of the paper in a collapsible format, has replaced the Supplementary information. You can submit up to 5 images as Expanded View. Please follow the nomenclature Figure EV1, Figure EV2 etc. The figure legend for these should be included in the main manuscript document file in a section called Expanded View Figure Legends after the main Figure Legends section. Additional Supplementary material should be supplied as a single pdf file labeled Appendix. The Appendix should have page numbers and needs to include a table of content on the first page (with page numbers) and legends for all content. Please follow the nomenclature Appendix Figure Sx, Appendix Table Sx etc. throughout the text, and also label the figures and tables according to this nomenclature.

For more details please refer to our guide to authors:
http://www.embopress.org/page/journal/14693178/authorguide#manuscriptpreparation

See our guide for figure preparation:
http://wol-prod-cdn.literatumonline.com/pb-assets/embo-site/EMBOPress_Figure_Guidelines_061115-1561436025777.pdf

See also the guidelines for figure legend preparation:
https://www.embopress.org/page/journal/14693178/authorguide#figureformat

3) a .docx formatted letter INCLUDING the reviewers' reports and your detailed point-by-point responses to their comments. As part of the EMBO Press transparent editorial process, the point-by-point response is part of the Review Process File (RPF), which will be published alongside your paper.

4) a complete author checklist, which you can download from our author guidelines (https://www.embopress.org/page/journal/14693178/authorguide). Please insert page numbers in the checklist to indicate where the requested information can be found in the manuscript. The completed author checklist will also be part of the RPF.

Please also follow our guidelines for the use of living organisms, and the respective reporting guidelines:
http://www.embopress.org/page/journal/14693178/authorguide#livingorganisms

5) that primary datasets produced in this study (e.g. RNA-seq, ChIP-seq and array data) are deposited in an appropriate public

database. This is now mandatory (like the COI statement). If no primary datasets have been deposited in any database, please state this in this section (e.g. 'No primary datasets have been generated and deposited').

See also: http://embor.embopress.org/authorguide#datadeposition

Please remember to provide a reviewer password if the datasets are not yet public.

The accession numbers and database should be listed in a formal "Data Availability " section (placed after Materials & Methods) that follows the model below. Please note that the Data Availability Section is restricted to new primary data that are part of this study.

Data availability

The datasets produced in this study are available in the following databases:

- RNA-Seq data: Gene Expression Omnibus GSE46843 (https://www.ncbi.nlm.nih.gov/geo/query/acc.cgi?acc=GSE46843)
- [data type]: [name of the resource] [accession number/identifier/doi] ([URL or identifiers.org/DATABASE:ACCESSION])

*** Note - All links should resolve to a page where the data can be accessed. ***

Moreover, I have these editorial requests:

6) We strongly encourage the publication of original source data with the aim of making primary data more accessible and transparent to the reader. The source data will be published in a separate source data file online along with the accepted manuscript and will be linked to the relevant figure. If you would like to use this opportunity, please submit the source data (for example scans of entire gels or blots, data points of graphs in an excel sheet, additional images, etc.) of your key experiments together with the revised manuscript. If you want to provide source data, please include size markers for scans of entire gels, label the scans with figure and panel number, and send one PDF file per figure.

7) Our journal encourages inclusion of *data citations in the reference list* to directly cite datasets that were re-used and obtained from public databases. Data citations in the article text are distinct from normal bibliographical citations and should directly link to the database records from which the data can be accessed. In the main text, data citations are formatted as follows: "Data ref: Smith et al, 2001" or "Data ref: NCBI Sequence Read Archive PRJNA342805, 2017". In the Reference list, data citations must be labeled with "[DATASET]". A data reference must provide the database name, accession number/identifiers and a resolvable link to the landing page from which the data can be accessed at the end of the reference. Further instructions are available at: http://www.embopress.org/page/journal/14693178/authorguide#referencesformat

8) Regarding data quantification and statistics, please specify, where applicable, the number "n" for how many independent experiments (biological or technical replicates) were performed, the bars and error bars (e.g. SEM, SD) and the test used to calculate p-values in the respective figure legends. Please provide statistical testing where applicable, and also add a paragraph detailing this to the methods section. See:
http://www.embopress.org/page/journal/14693178/authorguide#statisticalanalysis

9) Please note our reference format:
http://www.embopress.org/page/journal/14693178/authorguide#referencesformat

10) Please order the manuscript sections like this (using these names):
Title page - Abstract - Introduction - Results - Discussion - Materials and Methods -Data availability section - Acknowledgements - Author contributions - Conflict of interest statement - References - Figure legends - Expanded View Figure legends.

I look forward to seeing a revised version of your manuscript when it is ready. Please let me know if you have questions or comments regarding the revision.

Best,

Achim Breiling

Achim Breiling
Senior Editor
EMBO Reports

Referee #1:

In their study "IL-21 lowers the B cell receptor affinity threshold for participation in a T cell dependent immune response", Dvorsceket al study the role of IL21 signalling in the B cell entry to the germinal center reaction. The authors use an impressive set of mouse genetic tools to address their research question. Unfortunately, the experimental breadth and the novelty of the results remains shallow. A major part of the manuscript simply describes the defects in proliferation of the B cells lacking IL21. It is hard to see how much novel information these analyses bring to the field, as the role of IL21 in B cell proliferation and survival is well established. Also the signalling results can be questioned for their novelty. The last experiment, where different affinity HEL constructs are used, is interesting, yet again, only proliferation is used as a readout. No possible mechanistic information is provided. As the manuscripts is mainly based on observations of questionable impact and lacks mechanistic insights, it does not appear like a candidate to be published at EMBO Journal.

A comment on the data presentation. When data is only shown as ratios of WT/KO, a lot of information is hidden from the reader. This should be corrected.

Referee #2:

This is an interesting manuscript that addresses the role of IL-21 during the early phases of a T cell-dependent antibody response in vivo and finds that it preferentially acts to promote proliferation of lower affinity B cells. The key results are shown in Figure 5, and these data will likely have a significant impact on thinking in the field, in part given that the authors have used a very elegant in vivo experimental system, with likely relevance to authentic immune responses. The potential impact of these findings would be greater, however, if the connection to B cell biology, namely initiation of a germinal center (GC) response vs. contribution to the rapid extrafollicular antibody response (two alternative fates at this stage, along with B cell memory), were to be more explicitly determined (specific comment 1). The in vivo studies shown in the other figures (Fig. 1 and 2) are in general scientifically sound and add somewhat to the conclusions by indicating that IL-21 promotes cell cycling, possibly in multiple ways (shortening the cell cycle time, increasing entry into cell cycle). As these experiments are mostly done with the HEL2x antigen, but the effect of IL-21 is greatest with the HEL3x antigen, the effects shown in these figures are not huge, but probably big enough to explain the data for HEL2x presented in Fig. 5. Fig. 3 is also fine as it supports the conclusion of Fig. 2. Here, the system is more contrived, the authors are delivering IL-21 for a limited time window in the middle of the response by injecting it in a situation where endogenous IL-21 can't be made. Given that this is supporting data, it is OK that the system is somewhat artificial. Fig. 4 is an in vitro effort to address the signaling mechanism by which IL-21 promotes proliferation of B cells. These data implicate signaling downstream of PI 3-kinase/Akt as important, in agreement with some earlier work and known roles in proliferation in general. As with Fig. 3, the in vitro system is an imperfect match for the more physiological in vivo experiments. Here naïve B cells are stimulated with IL-21 at time 0 to look at signaling events and synergy with other stimuli (BCR and CD40), but in vivo, the BCR signal would likely be coming several hours or longer before the antigen-activated B cells migrated to the boundary to the T cell zone to interact with helper T cells (see comments 4 and 5 for how this could be improved). On the positive side, an effect on B cell proliferation is recapitulated, giving Fig. 4 some relevance to this work.

1. A significant limitation in this study that should be addressed either by additional experiments or by quoting relevant literature, if this has already been shown with this exact system (HEL-fused to ovalbumin as the antigen and using these particular BCR and TCR transgenic mice), is whether the various B cells (wild type or IL-21R-deficient) responding to the different antigens (HEL, HEL2x, HEL3x) are contributing to GC responses, extrafollicular antibody responses or both (and if both in what relative proportions). The authors have only analyzed the numbers of B cells and their numbers of proliferative cycles up to day 4.5, which is approximately when a GC response is initiating, and they have done nothing to characterize the B cells at this time with regard to their cell surface phenotype for GC markers, expression of BCL6, expression of BLIMP1, or numbers of plasmablasts. While these things have been looked at in the SW HEL system previously, typically the system is set up somewhat differently (using RBC conjugation of HEL, etc.) and in any case, it would be more compelling to have the data available here in this paper, with the analysis done fully in parallel rather than trying to compare to a previous study which may have unrecognized variables. Also, it is entirely possible that the fates of the B cells will vary depending on which affinity antigen is used.

2. As a result of the limitation described in comment 1, the Abstract and Summary are misleading in discussing relevance to GC responses, as this is likely, but not addressed here. In most places in the ms. and in the title, the authors use more appropriate language such as "Initial B cell response" and "T dependent immune response". Unless experiments are added to address comment 1, then the Abstract, Summary and also the Discussion need to be clearer about what are or are not the implications of the data presented (or why, based on earlier work a leap to GC responses is likely reasonable).

3. With regard to Fig. 4 C-E: it is good that the authors have realized that the primary data (4C) indicate that there are different fractions of B cells responding and to different extents and hence have analyzed the data with respect to two parameters (% of cells responding and MFI of the positive cells). Nonetheless, I think this still is not entirely the best way of communicating the actual results. The positively responding cells end up with the same or nearly same MFI values of phospho-S6, but what is changing is the fraction of cells that have an intermediate response (lower phospho-S6 but more positive than the unresponsive B cells). So some minor changes to the Results section here may be helpful in more accurately describing the results. Maybe the authors should represent the results as three categories: % unresponsive, % fully responsive, % intermediate responsive.

4. Also with regard to Fig. 4, phospho-S6 data in particular, are the times optimal in each stimulation? BCR secondary crosslinking is for 1 min only, the peak response might be later in some cases. As mentioned above, a more physiological relevant experiment might be to stimulate the cells with BCR crosslinking for some number of hours and then come in with IL-21 or anti-CD40. Staining with anti-phospho-STAT3 could perhaps be used to determine at what times in vivo there is IL-21 signaling happening in the B cells.
5. Also related to Fig. 4, previous studies have shown that HEL3x will stimulate signaling events in anti-HEL transgenic B cells, so these studies would be better connected to the rest of the paper if the authors used the SW HEL B cells and gave them the different affinity antigens with or without IL-21 and/or anti-CD40.

Referee #3:

The paper is the latest take on the role of IL-21 in B cell responses. It adds potentially interesting information about the contribution of IL-21 to the early, pre-GC expansion of antigen-specific B cells. The paper uses and excellent use of mouse genetic tools (the swHEL Tg on Rag1-/- background, IL-21R -/-) along with novel mutated HEL-OVA antigens to evaluate responses of transferred, HEL-specific B and OVA-specific T cells and their dependence on antigen affinity. The conclusion derived from these studies is that IL-21 promotes proliferation of B cells before the GC onset and that this has a stronger effect on low-affinity B cells compared to high-affinity B cells, allowing their better expansion. Previously, preGC expansion has been shown to be competitive in terms of B cell affinity and thus the modulation of the selection by IL-21 is interesting.
Overall, the experiments are well done and carefully analyzed and cleanly described. The point is simple, but potentially adds a worthwhile knowledge to the spectrum of effects that IL-21 has in the B cell responses.

Strong points:
- demonstration of effects on IL-21 on the cell cycle of activated B cells
- demonstration of additive effects with BCR and CD40 signalling
- clean in vivo system to track responses of antigen-specific B cells responding with varying affinity to engineered HEL antigens
- shows effects of IL-21 on pre-GC B cell proliferation, with strongest effect at weak BCR affinity

Major critiques:

The main point hinges on just one in vivo experiment. While it is true that the effects on IL-21 were strongest on the weakest of HEL affinities, it is a quantitative effect and thus the relationship of affinity and the requirement for IL-21 should be explored in more detail with more evidence.

For the in vivo results, the analysis is detailed, but quite complicated and focuses on the proliferation. What were the final cell number of the GC cells at the onset of the GC reaction?

Since the effects of IL-21 are quantitative, and the signalilling additive with CD40, it is not clear why at lower BCR affinity there would be a qualitative step up in the requirement for IL-21. The in vitro titration of anti-CD40 does not quite mimic the in vivo data comparing different antigen affinities. Can the in vitro experiments be more realistic, for example a T cell-dependent B cell proliferation assays using the HEL-OVA system? This way the effect of different IL-21 concentrations on B cells stimulated with different amount of T cell help could be measured (eg. using IL-21 -/- OT-II T cells supplemented or not with IL-21). This could resolve where exactly IL-21 provides the strongest effect.

The additive effects of the different stimuli on pS6 are interesting, however, it is not directly clear from the experiments whether this pathway is important/limiting for the B cell proliferation in this system.

Minor points:
How are the HEL-biotin antigens stained on the surface of the cells in Figure S1? Are these HEL-streptavidin tetramers, or is streptavidin used as a secondary staining?

Are the IL-21R -/- GFP- B cells also rag1-/-? It is not entirely clear from the schematics.

On page 6 the text states that "Phosphorylation of S6 was minimally induced following CD40 ligation and more so following BCR stimulation but potently increased by the presence of IL-21 (Fig. 4C)" . It seems however that pS6 is much better induced by CD40 than by the BCR.

Before addressing the specific referees' points, we have to explain the relatively substantial change in the manuscript. As part of reappraising the work for this submission, we carefully re-analyzed all our experiments and realized that some data in Figure 5 were the result of an artefact in gating. What we concluded was the complete dilution of CTV by extensive proliferation of a fraction of WT B cells in response to $HEL^{3x}OVA_{pep}$ immunization was in fact due to a gating omission that saw a small number of errant cells incorrectly included as SW_{HEL} B cells. This problem did not occur in *Il21*$^{-/-}$ SW_{HEL} B cells as they were additionally identified as transgenic for GFP. While this necessitated removing the conclusion of IL-21 having a role in recruiting low-affinity B cells into the response, we are confident of the validity of everything that is now included. This is because we have:

1) re-analyzed all data using a revised gating strategy that completely excludes spurious events;
2) not drawn any conclusions from rare events in the immunized mice and
3) performed experiments analyzing the response on day 7 post immunization when a robust GC immune response is present in all immunization conditions with results that confirm and extend our conclusions.

Unfortunately, the now removed erroneous conclusion was one of some significance and contributed to the title and the abstract. Both have been amended to reflect only those conclusions of which we are certain. We now conclude that IL-21 amplifies the expansion of B cells regardless of BCR affinity, and additionally, that IL-21 increases PC output from GC by promoting the overall response rather than PC differentiation directly. We apologize unreservedly for this mistake and are confident in the robustness of the conclusions presented in this revised version. Furthermore, we hope that we have addressed all remaining substantive concerns raised by the referees about the initial submission, and that the work meets the standards required for acceptance. Finally, while one conclusion has necessarily changed, we remain convinced that the work is novel and significant and will greatly enhance understanding of how IL-21 regulates the B cell response to antigenic challenge at both cellular and molecular levels.

Sincerely,

Isaak Quast

Referee #1:

In their study "IL-21 lowers the B cell receptor affinity threshold for participation in a T cell dependent immune response", Dvorsceket al study the role of IL21 signalling in the B cell entry to the germinal center reaction. The authors use an impressive set of mouse genetic tools to address their research question. Unfortunately, the experimental breadth and the novelty of the results remains shallow. A major part of the manuscript simply describes the defects in proliferation of the B cells lacking IL21. It is hard to see how much novel information these analyses bring to the field, as the role of IL21 in B cell proliferation and survival is well established. Also the signalling results can be questioned for their novelty. The last experiment, where different affinity HEL constructs are used, is interesting, yet again, only proliferation is used as a readout. No possible mechanistic information is provided. As the manuscripts is mainly based on observations of questionable impact and lacks mechanistic insights, it does not appear like a candidate to be published at EMBO Journal.

A comment on the data presentation. When data is only shown as ratios of WT/KO, a lot of information is hidden from the reader. This should be corrected.

While IL-21 has indeed been shown to promote proliferation, here we show how this applies *in vivo* and reveal a role for IL-21 well before onset of the germinal center (GC) response. Our cell cycle analysis shows that IL-21 modulates both entry and duration of the cell cycle, information we consider novel and conceptually crucial to understand the effects of IL-21 in humoral immunity. Similarly, while the signaling downstream of the IL-21R has been characterized, we now show IL-21R signaling intersects with and augments BCR and CD40 signaling, thus providing key insights into its function in B cell activation and selection. In our day 4.5 experiment (Fig. 5), we provide a detailed analysis of the dependency of B cell activation on BCR affinity and characterize the expression of molecules associated with B cell differentiation across individual cell divisions. We have additionally complemented this analysis with a phenotypic B cell analysis, including GC and PC differentiation on day 7 post immunization. These experiments show that IL-21's primary role in promoting PC differentiation is by initiating and sustaining B cell proliferation rather than altering the balance in the bifurcation between GC B cell and PC fates. In summary, we would respectfully argue that our results significantly advance the understanding of B cell

activation *in vivo* by revealing a crucial role for IL-21 in recruitment of B cells into the GC response and by identifying it as a ubiquitous response amplifier rather than a mediator of selection or differentiation.

Referee #2:

This is an interesting manuscript that addresses the role of IL-21 during the early phases of a T cell-dependent antibody response in vivo and finds that it preferentially acts to promote proliferation of lower affinity B cells. The key results are shown in Figure 5, and these data will likely have a significant impact on thinking in the field, in part given that the authors have used a very elegant in vivo experimental system, with likely relevance to authentic immune responses. The potential impact of these findings would be greater, however, if the connection to B cell biology, namely initiation of a germinal center (GC) response vs. contribution to the rapid extrafollicular antibody response (two alternative fates at this stage, along with B cell memory), were to be more explicitly determined (specific comment 1). The in vivo studies shown in the other figures (Fig. 1 and 2) are in general scientifically sound and add somewhat to the conclusions by indicating that IL-21 promotes cell cycling, possibly in multiple ways (shortening the cell cycle time, increasing entry into cell cycle). As these experiments are mostly done with the HEL2x antigen, but the effect of IL-21 is greatest with the HEL^{3x} antigen, the effects shown in these figures are not huge, but probably big enough to explain the data for HEL^{2x} presented in Fig. 5. Fig. 3 is also fine as it supports the conclusion of Fig. 2. Here, the system is more contrived, the authors are delivering IL-21 for a limited time window in the middle of the response by injecting it in a situation where endogenous IL-21 can't be made. Given that this is supporting data, it is OK that the system is somewhat artificial. Fig. 4 is an in vitro effort to address the signaling mechanism by which IL-21 promotes proliferation of B cells. These data implicate signaling downstream of PI 3-kinase/Akt as important, in agreement with some earlier work and known roles in proliferation in general. As with Fig. 3, the in vitro system is an imperfect match for the more physiological in vivo experiments. Here naïve B cells are stimulated with IL-21 at time 0 to look at signaling events and synergy with other stimuli (BCR and CD40), but in vivo, the BCR signal would likely be coming several hours or longer before the antigen-activated B cells migrated to the boundary to the T cell zone to interact with helper T cells (see comments 4 and 5 for how this could be improved). On the positive side, an effect on B cell proliferation is recapitulated, giving Fig. 4 some relevance to this work.

1. A significant limitation in this study that should be addressed either by additional experiments or by quoting relevant literature, if this has already been shown with this exact system (HEL-fused to ovalbumin as the antigen and using these particular BCR and TCR transgenic mice), is whether the various B cells (wild type or IL-21R-deficient) responding to the different antigens (HEL^{WT}, HEL^{2x}, HEL^{3x}) are contributing to GC responses, extrafollicular antibody responses or both (and if both in what relative proportions). The authors have only analyzed the numbers of B cells and their numbers of proliferative cycles up to day 4.5, which is approximately when a GC response is initiating, and they have done nothing to characterize the B cells at this time with regard to their cell surface phenotype for GC markers, expression of BCL6, expression of BLIMP1, or numbers of plasmablasts. While these things have been looked at in the SW_{HEL} system previously, typically the system is set up somewhat differently (using RBC conjugation of HEL, etc.) and in any case, it would be more compelling to have the data available here in this paper, with the analysis done fully in parallel rather than trying to compare to a previous study which may have unrecognized variables. Also, it is entirely possible that the fates of the B cells will vary depending on which affinity antigen is used.

There are two points in this comment:

1.1 Contribution of SW_{HEL} B cells to GC and PC response: By repeating the experiment shown in Figure 5 but now analyzing on day 7, we confirm the contribution of SW_{HEL} B cells to the GC response in all immunization conditions (NEW Figure 6A-C) as indicated by co-expression of the characteristic GC B cell markers GL7 and Fas (NEW Figure EV4B) and CD86 and CXCR4 (NEW Figure EV4C). As expected, plasmablast/plasma cell differentiation was affinity-dependent and only consistently observed upon HEL^{WT} and HEL^{2x}-OVA_{pep} immunization (NEW Figure 6D).

1.2 Characterizing the phenotype of SW_{HEL} B cells on day 4.5: The original experiments actually included the staining for molecules known to be dynamically regulated during early B cell activation - IgD, CD38, Fas and CD138. We agree that providing this analysis is crucial. At the same time, it is important to note that early differentiation is tightly linked to the number of cell divisions a B cell undergoes. Thus, we decided to analyze the expression of these markers relative to CTV dilution, which

required us pooling all mice in an experiment to give sufficient cell numbers for a robust analysis. As shown in NEW Figure 5F, the changes in expression of these molecules are highly similar between WT and IL-21R deficient SW_{HEL} B cells and tightly correlated with the number of divisions a cell has undergone.

2. As a result of the limitation described in comment 1, the Abstract and Summary are misleading in discussing relevance to GC responses, as this is likely, but not addressed here. In most places in the ms. and in the title, the authors use more appropriate language such as "Initial B cell response" and "T dependent immune response". Unless experiments are added to address comment 1, then the Abstract, Summary and also the Discussion need to be clearer about what are or are not the implications of the data presented (or why, based on earlier work a leap to GC responses is likely reasonable).

Our additional analysis now confirms that the responses we analyze on days 2.5-4.5 transpire into mature GC.

3. With regard to Fig. 4 C-E: it is good that the authors have realized that the primary data (4C) indicate that there are different fractions of B cells responding and to different extents and hence have analyzed the data with respect to two parameters (% of cells responding and MFI of the positive cells). Nonetheless, I think this still is not entirely the best way of communicating the actual results. The positively responding cells end up with the same or nearly same MFI values of phospho-S6, but what is changing is the fraction of cells that have an intermediate response (lower phospho-S6 but more positive than the unresponsive B cells). So some minor changes to the Results section here may be helpful in more accurately describing the results. Maybe the authors should represent the results as three categories: % unresponsive, % fully responsive, % intermediate responsive.

We have analyzed the data as suggested by the referee (PBP Response Figure 1 below) and while this re-affirms our findings, we think little additional information is gained. Our preference is to not include it in the manuscript for the sake of brevity but we are happy to do so if the referee feels it is essential.

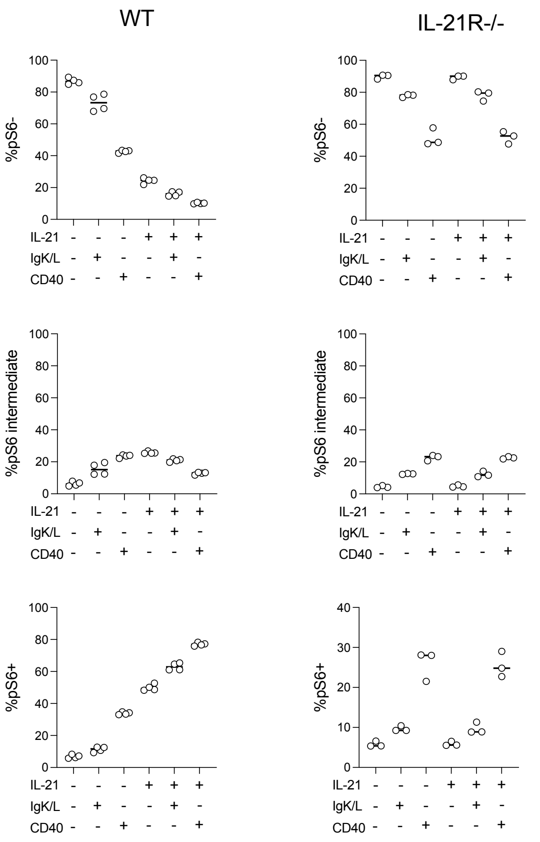

PBP Response Figure 1:
Re-analysis of data shown in Figure 4D by gating on pS6 negative, intermediate and high/+ cells.

4. Also with regard to Fig. 4, phospho-S6 data in particular, are the times optimal in each stimulation? BCR secondary crosslinking is for 1 min only, the peak response might be later in some cases. As

mentioned above, a more physiological relevant experiment might be to stimulate the cells with BCR crosslinking for some number of hours and then come in with IL-21 or anti-CD40. Staining with anti-phospho-STAT3 could perhaps be used to determine at what times in vivo there is IL-21 signaling happening in the B cells.

There are three aspects to point 4:

4.1 Optimal timing of signaling analysis: While the time points were chosen based on published literature (Luo et a 2018, Immunity Wensveen et al. 2016, Scientific Reports), the reviewer raises an important point and we have now performed a longitudinal analysis of p-AKT and p-S6 following BCR cross-linking in the presence or absence of IL-21. As shown in new NEW Fig. EV2B-D, the majority of the BCR-mediated effect for both p-AKT and p-S6 occurs within the first minute with p-S6 increasing further up to 15 minutes post stimulation. Importantly, these experiments highlighted the sustained effect of IL-21 on p-AKT and p-S6 and re-iterated the additive nature BCR and IL-21 signaling.

4.2 Physiological sequence of signaling events: We agree that antigen capture, and BCR cross-linking are likely the first events in B cell activation and IL-21 may either be present at this time or not. To address if the role of IL-21 is related to the sequence of signaling events, we incubated naive B cells for 20 min with anti-IgK/L followed by avidin-mediated cross-linking for 30 minutes before addition of IL-21 and analysis 1.5 hours thereafter. For conditions including anti-CD40, it was added during the final hour of incubation. As shown in NEW Figures EV2E-G, BCR cross-linking resulted in minimal p-S6 or p-AKT, presumably due to the extended incubation period (2 hours following avidin addition). However, IL-21 again additively amplified p-AKT and p-S6 if present in conjunction with anti-CD40. We conclude from these experiments that the effect of IL-21 is independent of whether it is present before or after BCR stimulation.

4.3: *In vivo* signaling events. We agree that knowledge of the exact timing of IL-21 signaling *in vivo* would be interesting, however, given the complexity of *in vivo* systems (short pSTAT3 half-life, non-synchronous encounter of antigen and T cell help, multiple cytokines resulting in STAT3 phosphorylation) studying this is extremely challenging and we consider it beyond the scope of this manuscript.

5. Also related to Fig. 4, previous studies have shown that HEL3x will stimulate signaling events in anti-HEL transgenic B cells, so these studies would be better connected to the rest of the paper if the authors used the SW HEL B cells and gave them the different affinity antigens with or without IL-21 and/or anti-CD40.

We think the reviewer is referring to Wensveen et al., 2016. In our hands, monomeric HEL-OVA proteins do not result in p-S6 or pAKT. We have re-assessed the possibility of replicating the data shown in Figure 4 with $HEL^{WT}OVA_{pep}$ and $HEL^{3X}OVA_{pep}$ and cross-linking the SW_{HEL} BCR by adding a monoclonal anti-HEL antibody that does not compete with the SW_{HEL} BCR (clone HyHEL9). As shown in PBP Response Figure 2, we were able to see some p-S6 and pAKT signal upon $HEL^{WT}OVA_{pep}$ and HyHEL9 co-incubation with IL-21 again additively increasing pS-6 and pAKT. However, we did not see any phosphorylation of either protein when using $HEL^{3X}OVA_{pep}$ making it very difficult if not impossible for us to study the effect of affinity using HEL variants *in vitro*. Instead, we chose to investigate the role of BCR affinity indirectly, by assessing the effect of limiting T cell help (replicated by anti-CD40 titration, Figure 4F), access to which *in vivo* is dependent on the efficiency of antigen capture and thus BCR affinity. To further strengthen these data, we have performed additional experiments and include these together with statistical analysis of the data (NEW Figure 4G).

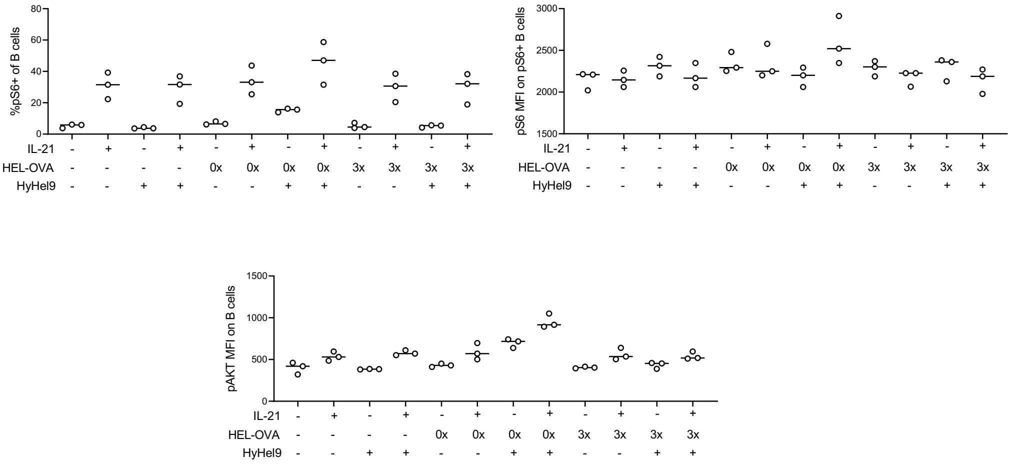

PBP Response Figure 2:
SW_{HEL} B cells were incubated in vitro with IL-21 (20 ng/ml) for 3 hours followed by HEL-OVA (10 µg/ml) and/or HyHel9 (1 µg/ml) for 30 minutes and analysed by phosphoflow detecting pS6 (upper panels) and pAKT (lower panel).

Referee #3:

The paper is the latest take on the role of IL-21 in B cell responses. It adds potentially interesting information about the contribution of IL-21 to the early, pre-GC expansion of antigen-specific B cells. The paper uses and excellent use of mouse genetic tools (the Tg on *Rag1*$^{-/-}$ background, IL-21R $^{-/-}$) along with novel mutated HEL-OVA antigens to evaluate responses of transferred, HEL-specific B and OVA-specific T cells and their dependence on antigen affinity. The conclusion derived from these studies is that IL-21 promotes proliferation of B cells before the GC onset and that this has a stronger effect on low-affinity B cells compared to high-affinity B cells, allowing their better expansion. Previously, preGC expansion has been shown to be competitive in terms of B cell affinity and thus the modulation of the selection by IL-21 is interesting.
Overall, the experiments are well done and carefully analyzed and cleanly described. The point is simple, but potentially adds a worthwhile knowledge to the spectrum of effects that IL-21 has in the B cell responses.

Strong points:
- demonstration of effects on IL-21 on the cell cycle of activated B cells
- demonstration of additive effects with BCR and CD40 signalling
- clean in vivo system to track responses of antigen-specific B cells responding with varying affinity to engineered HEL antigens
- shows effects of IL-21 on pre-GC B cell proliferation, with strongest effect at weak BCR affinity

Major critiques:

The main point hinges on just one in vivo experiment. While it is true that the effects on IL-21 were strongest on the weakest of HEL affinities, it is a quantitative effect and thus the relationship of affinity and the requirement for IL-21 should be explored in more detail with more evidence.

We completely agree with this assessment and as outlined in our introductory statement, we have re-analyzed our data and carefully re-considered our conclusions to ensure they are based on solid experimental data.

For the in vivo results, the analysis is detailed, but quite complicated and focuses on the proliferation. What were the final cell number of the GC cells at the onset of the GC reaction?

We have now reduced the number of analyses to those addressing proliferation, focusing solely on cell numbers in CTV division peaks (NEW Figure 5 E) and showing the events in the division peaks of

pooled cells as absolute counts rather than modal, to display a more complete and detailed picture (NEW Figure 5D). By analyzing the response on day 7, we now additionally provide numbers of GC B cells and PC (NEW Figure 6).

Since the effects of IL-21 are quantitative, and the signalling additive with CD40, it is not clear why at lower BCR affinity there would be a qualitative step up in the requirement for IL-21. The in vitro titration of anti-CD40 does not quite mimic the in vivo data comparing different antigen affinities. Can the in vitro experiments be more realistic, for example a T cell-dependent B cell proliferation assays using the HEL-OVA system? This way the effect of different IL-21 concentrations on B cells stimulated with different amount of T cell help could be measured (eg. using IL-21 -/- OT-II T cells supplemented or not with IL-21). This could resolve where exactly IL-21 provides the strongest effect.

Please excuse the poor description of our experimental rationale and we now provide further detail. Our rationale for using anti-CD40 titration as a surrogate for HEL-OVA affinity variants was: BCR affinity is directly proportional to the efficiency of antigen uptake, which will in turn determine the ability to elicit T cell help, with CD40 being the key (albeit not only) constituent of T cell help. Thus, by titrating anti-CD40 in the presence or absence of IL-21, we mimic the affinity-dependent access to T cell help and can thus study if the importance of IL-21 depends on the extent of CD40 signaling. On the other hand, using the different HEL-OVA affinity variants *in vitro* is complicated by us not being able to replicate competition as well as adjuvant-mediated and antibody-mediated immune complex formation. Also, adding IL-21 to IL-21-deficient T/B cell cultures to study B cell intrinsic functions is complicated by the key role of IL-21 in T cell effector differentiation. We would thus argue, with respect, that using affinity variants *in vitro* will not bring us closer to what occurs *in vivo*.

Despite these limitations, we think our data clearly indicate that IL-21 augments key signaling pathways downstream of BCR and CD40, which are likely to have consequences for B cell expansion *in vivo*. However, we agree with the reviewer that the limitations of our *in vitro* findings for their applicability *in vivo* should be discussed in the manuscript and we have made relevant changes.

We have now further clarified our experimental rationale:

Results:

Page 7: "T cell help via CD40 ligation is essential for TD immune responses *in vivo [42]*, and B cell access to T cell help - and thus CD40 signaling - is determined in large part by BCR affinity [12, 43]. The additive effect of IL-21 on B cell signaling suggested that IL-21 could have influenced this relationship by adjusting the minimal CD40 signaling threshold and/or by amplifying the response to CD40 ligation."

Page 7: "The increased initiation and expedited progression through division in response to IL-21 and the amplification of p-S6 signaling suggested a role for IL-21 in the recruitment of B cells into GC responses. Moreover, with T cell help to B cells being determined by the BCR affinity-dependent efficiency of antigen capture and presentation in the context of MHC-II [12, 43], the increased cell cycle initiation at low anti-CD40 concentrations in the presence of IL-21 *in vitro* suggested that IL-21 may have influenced the entry and continued participation of B cells in a BCR affinity-dependent manner."

Discussion:

Page 10: "Our *in vitro* experiments, while not directly assessing BCR affinity-dependent signaling, imply that the *in vivo* consequences of IL-21 on early B cell activation are – at least in part – a result of the additive nature of BCR, CD40 and IL-21R on AKT and S6 phosphorylation."

The additive effects of the different stimuli on pS6 are interesting, however, it is not directly clear from the experiments whether this pathway is important/limiting for the B cell proliferation in this system.

We agree that the exact role of S6 in B cells remains poorly understood and we do not directly prove the requirement of S6 in the IL-21 dependent effects observed. However, given the potent S6 phosphorylation upon IL-21 exposure of naïve B cells *in vitro* and together with AKT and S6 being part of the mTOR signaling network - a key regulator of cell growth and proliferation - we consider a role for S6 in the proliferation-enhancing function of IL-21 highly likely. We discuss this in more detail, highlighting the limitations of our study and citing additional literature:

Discussion, page 10:

“While surprisingly little is known about the specific function of S6 in B cells, both AKT and S6 are part of the mTOR signaling network, a key regulator of cell metabolism and proliferation (reviewed [50]). In addition, B cell proliferation, particularly in response to BCR signaling, is highly sensitive to rapamycin, an mTOR inhibitor that also inhibits S6 phosphorylation [51, 52]. Thus, we consider it likely that the convergence of proliferation-inducing pathways on p-S6 allows IL-21 to amplify the B cell response by increasing the basal p-S6 amount thereby facilitating B cell activation and proliferation in conjunction with BCR or CD40 signaling.”

Discussion, page 10:

“Our *in vitro* experiments, while not directly assessing BCR affinity-dependent signaling, imply that the *in vivo* consequences of IL-21 on early B cell activation are – at least in part – a result of the additive nature of BCR, CD40 and IL-21R on AKT and S6 phosphorylation. In support of this, p-S6 was found to be highly enriched in GC B cells expressing c-Myc [58], a population of cells about to enter cell division as a consequence of receiving T cell help [59].”

Minor points:
How are the HEL-biotin antigens stained on the surface of the cells in Figure S1? Are these HEL-streptavidin tetramers, or is streptavidin used as a secondary staining?

Streptavidin is used as a secondary staining. We have now included a description in the methods section.

Materials & Methods, page 13:

“In experiments where antigen-binding was analyzed, cells were first incubated with 400 ng/mL biotinylated $HEL^{WT}OVA_{pep}$, $HEL^{2X}OVA_{pep}$ or $HEL^{3X}OVA_{pep}$ in staining buffer on ice for 30 minutes, then washed once and then incubated with monoclonal antibodies and fluorochrome-conjugated streptavidin.”

Are the *IL-21R* $^{-/-}$ GFP- B cells also *rag1*$^{-/-}$? It is not entirely clear from the schematics.

Yes, both are Rag$^{-/-}$, we have updated the manuscript for clarity.

Results, page 3:

“WT or *Il21r*$^{-/-}$ mice were crossed with mice that carried a knock-in rearranged BCR specific for hen egg lysozyme (BCR-HEL), known as SW_{HEL} mice [30, 31]. Additionally, these mice were crossed with *eGFP* transgenic mice and all WT or *Il21r*$^{-/-}$ SW_{HEL} mice used in this study were *Rag1* deficient, preventing endogenous BCR rearrangement during development and thus unintended B cell activation.”

On page 6 the text states that "Phosphorylation of S6 was minimally induced following CD40 ligation and more so following BCR stimulation but potently increased by the presence of IL-21 (Fig. 4C)" . It seems however that pS6 is much better induced by CD40 than by the BCR.

We are sorry for the mistake and have corrected the manuscript accordingly.

Results, page 6:

“Phosphorylation of S6 was minimally induced following BCR stimulation and more so following CD40 ligation but potently increased by the presence of IL-21 (Fig. 4C).”

Dear Dr. Quast,

Thank you for the submission of your revised manuscript to our editorial offices. I have now received reports from the two referees that were asked to re-evaluate your study, you will find below. As you will see, both referees now support the publication of your study in EMBO reports. Both referees have some remaining points and suggestions, I ask you to address in a final revised manuscript. Please also provide a detailed final p-b-p-response to the remaining concerns of the referees.

Moreover, I have these editorial requests:

- We updated our journal's competing interests policy in January 2022 and request authors to consider both actual and perceived competing interests. Please review the policy https://www.embopress.org/competing-interests and update your competing interests if necessary. Please name this section 'Disclosure and Competing Interests Statement' and put it after the Acknowledgements section.

- The Figure legends are often very short and cryptic, which renders them difficult to comprehend for non-specialized readers. Please expand these, explaining in more detail what is shown and how the data were obtained.

- Please make sure that the number "n" for how many independent experiments were performed, their nature (biological versus technical replicates), the bars and error bars (e.g. SEM, SD) and the test used to calculate p-values is indicated in the respective figure legends (main, EV and Appendix figures), and that statistical testing has been done where applicable. Please avoid phrases like 'independent experiment', but clearly state if these were biological or technical replicates. Please add complete statistical testing to all diagrams (for main, EV and Appendix figures). Please also indicate (e.g. with n.s.) if testing was performed, but the differences are not significant.

- Please format the references according to our journal style. See:
http://www.embopress.org/page/journal/14693178/authorguide#referencesformat

- Please add a formal 'Data Availability Section' after the Methods. This is now mandatory, like the conflict of interest statement. If no primary datasets have been deposited, please state this section (e.g. 'No primary datasets have been generated and deposited').

- We now use CRediT to specify the contributions of each author in the journal submission system. CRediT replaces the author contribution section. Please use the free text box to provide more detailed descriptions. See also guide to authors:
https://www.embopress.org/page/journal/14693178/authorguide#authorshipguidelines

- There seems to be no callout for Fig. 6F in the manuscript text. Please check.

- Please move Table 1 to the end of the manuscript text and provide a legend.

- Finally, please find attached a word file of the manuscript text (provided by our publisher) with changes we ask you to include in your final manuscript text, and some queries, we ask you to address. Please provide your final manuscript file with track changes, in order that we can see any modifications done.

In addition, I would need from you:
- a short, two-sentence summary of the manuscript (around 35 words).
- three to four short bullet points highlighting the key findings of your study
- a schematic summary figure (in jpeg or tiff format with the exact width of 550 pixels and a height of not more than 400 pixels) that can be used as a visual synopsis on our website.

I look forward to seeing the final revised version of your manuscript when it is ready. Please let me know if you have questions regarding the revision.

Please use this link to submit your revision: https://embor.msubmit.net/cgi-bin/main.plex

Best,

Achim Breiling
Senior Editor
EMBO Reports

Referee #1 (referee #2 TEJ):

In this revised manuscript, the authors have addressed the question of the role of IL-21 in B cells during an antibody response to a protein antigen for which T cell help is required. Helper T cells release IL-21 as one of the cytokines that contributes to antibody responses, as has been shown previously. For the most part, the authors have addressed reasonably well my previous comments (Referee #2). To a significant extent, this was done by adding new and informative data. In particular, the responses to my comment #1 (new Fig 6; new Fig. 5F, etc.) fill in a gap that I think adds significantly to what this work will contribute to this topic by connecting better the pre-germinal center data (unique to this study) to the early germinal center response in the same experimental system. Several earlier papers looked at germinal centers with similar but distinct experimental systems, so this addition will allow people to fit this work into the previous observations with greater clarity. The conclusions are somewhat similar to earlier studies (namely that IL-21 promotes B cell proliferation), but takes this insight to an earlier stage of the response. So, in summary, I think this study adds something of value to the field by examining earlier stages of the response and also by delving into the issues more mechanistically to show that the effects are essentially limited to proliferation, both cell cycle entry and more rapid division time, and do not really alter survival or terminal differentiation fate choice.
Having said that, the revised manuscript has become less interesting in one feature: further analysis has caused the authors to remove of the conclusions present previously; they now find no obvious effect of BCR affinity for antigen on the ability of IL-21 to promote B cell proliferation during the pre-germinal center phase of the antibody response, whereas previously they thought that there was an effect. There is still a conclusion here but it is now a "negative result". In my opinion this is still a significant conclusion for the field, as other aspects of antibody responses do show important effects of BCR affinity.
In addition to in vivo studies with an elegantly engineered system, the manuscript also contains in vitro studies that examine the possible role of a subset of IL-21R signaling events, those related to PIP3 elevation, namely Akt activation and phosphorylation of ribosomal protein S6. These studies add something by showing that IL-21 boosts these signaling events more strongly than BCR or CD40 engagement (another signal provided by helper T cells). Having said that, as pointed out in the previous review, the in vitro experimental system is not well connected to the in vivo system used, limiting the ability to put the conclusions together. Moreover, the in vitro signaling studies are correlative in nature, there are no experiments presented that test the role of these signaling events. While Akt activation is almost certainly contributing to B cell proliferation, this issue is not addressed experimentally. For S6 phosphorylation, the uncertainly is greater still, as this signaling event is thought to regulate the translation rate for a subset of mRNAs, but its role in B cells is little studied. S6 phosphorylation is a highly robust signaling readout, and for this reason is worth including, but its role in B cell proliferation is not addressed. For these reasons, my one specific suggestion for improving the revised manuscript is that the authors should reword the Abstract to avoid misleading the reader into thinking there is a test of the importance of these signaling events for the biological events being studied (repeated below as "specific comment").

Specific comment:
1. The 4th sentence of the Abstract ("By activating AKT and S6, IL-6 accelerates ...") is misleading as it implies that the impact of AKT and S6 on B cell proliferation promoted by IL-21 have been assessed in this work, and that is not the case. The part starting with "IL-6 accelerates" to the end of the sentence is fine, but if the authors are going to mention AKT and S6, then the most that should be claimed is that the effect of IL-21 on these signaling events correlates with the effects on proliferation. Whether that can be done within the character limits, I don't know, but the Abstract should not claim to show something that is not actually shown, of which the current phrasing is guilty. I have little doubt that Akt is important for B cell proliferation, but it is not experimentally addressed here and the role of S6 is certainly not clear, it is a very robust readout for this signaling pathway, but the biological role in B cells and many other cell types is poorly understood.

Referee #2 (Referee #3 TEJ):

Despite the altered conclusions, this manuscript is cleaner and more consistent and generally addresses my previous concerns. The message is a bit more limited than the previous version, but it does have its significance.

My only comment is that I think the authors should extend the discussion of the similarities and differences from the previous paper looking at IL-21 signalling in B cells (Zotos et al 2010). These previous experiments used a non-competitive system as compared to the competitive transfers used here and they found little effect on total numbers of antigen-specific B cells, but reduced numbers of plasma cells and increased numbers of non-GC B cells. Some explanation for the reader of why the current manuscript differs from these previous studies would help to align this new paper with the literature.

Point-by-point response to referee comments.

Referee #1 (referee #2 TEJ):

In this revised manuscript, the authors have addressed the question of the role of IL-21 in B cells during an antibody response to a protein antigen for which T cell help is required. Helper T cells release IL-21 as one of the cytokines that contributes to antibody responses, as has been shown previously. For the most part, the authors have addressed reasonably well my previous comments (Referee #2). To a significant extent, this was done by adding new and informative data. In particular, the responses to my comment #1 (new Fig 6; new Fig. 5F, etc.) fill in a gap that I think adds significantly to what this work will contribute to this topic by connecting better the pre-germinal center data (unique to this study) to the early germinal center response in the same experimental system. Several earlier papers looked at germinal centers with similar but distinct experimental systems, so this addition will allow people to fit this work into the previous observations with greater clarity. The conclusions are somewhat similar to earlier studies (namely that IL-21 promotes B cell proliferation), but takes this insight to an earlier stage of the response. So, in summary, I think this study adds something of value to the field by examining earlier stages of the response and also by delving into the issues more mechanistically to show that the effects are essentially limited to proliferation, both cell cycle entry and more rapid division time, and do not really alter survival or terminal differentiation fate choice.
Having said that, the revised manuscript has become less interesting in one feature: further analysis has caused the authors to remove of the conclusions present previously; they now find no obvious effect of BCR affinity for antigen on the ability of IL-21 to promote B cell proliferation during the pre-germinal center phase of the antibody response, whereas previously they thought that there was an effect. There is still a conclusion here but it is now a "negative result". In my opinion this is still a significant conclusion for the field, as other aspects of antibody responses do show important effects of BCR affinity.
In addition to in vivo studies with an elegantly engineered system, the manuscript also contains in vitro studies that examine the possible role of a subset of IL-21R signaling events, those related to PIP3 elevation, namely Akt activation and phosphorylation of ribosomal protein S6. These studies add something by showing that IL-21 boosts these signaling events more strongly than BCR or CD40 engagement (another signal provided by helper T cells). Having said that, as pointed out in the previous review, the in vitro experimental system is not well connected to the in vivo system used, limiting the ability to put the conclusions together. Moreover, the in vitro signaling studies are correlative in nature, there are no experiments presented that test the role of these signaling events. While Akt activation is almost certainly contributing to B cell proliferation, this issue is not addressed experimentally. For S6 phosphorylation, the uncertainly is greater still, as this signaling event is thought to regulate the translation rate for a subset of mRNAs, but its role in B cells is little studied. S6 phosphorylation is a highly robust signaling readout, and for this reason is worth including, but its role in B cell proliferation is not addressed. For these reasons, my one specific suggestion for improving the revised manuscript is that the authors should reword the Abstract to avoid misleading the reader into thinking there is a test of the importance of these signaling events for the biological events being studied (repeated below as "specific comment").

Specific comment:
1. The 4th sentence of the Abstract ("By activating AKT and S6, IL-6 accelerates ...") is misleading as it implies that the impact of AKT and S6 on B cell proliferation promoted by IL-21 have been assessed in this work, and that is not the case. The part starting with "IL-6 accelerates" to the end of the sentence is fine, but if the authors are going to mention AKT and S6, then the most that should be claimed is that the effect of IL-21 on these signaling events correlates with the effects on proliferation. Whether that can be done within the character limits, I don't know, but the Abstract should not claim to show something that is not actually shown, of which the current phrasing is guilty. I have little doubt that Akt is important for B cell proliferation, but it is not experimentally addressed here and the role of S6 is certainly not clear, it is a very robust readout for this signaling pathway, but the biological role in B cells and many other cell types is poorly understood.

We agree with Referee 1 and have now adjusted the manuscript text to accurately reflect our results:

Page1, Abstract:

Here we report how the signature cytokine of follicular helper T cells, IL-21, acts as a key regulator of the initial B cell response. By activating AKT and S6, IL-21 accelerates cell cycle progression and the rate of cycle entry of B cells, increasing their contribution to the ensuing GC. This effect occurs over a wide range of initial B cell receptor affinities and the resultant increased proliferation can explain the IL-21-mediated promotion of plasma cell differentiation.

Was changed to:

Here we report how the signature cytokine of follicular helper T cells, IL-21, acts as a key regulator of the initial B cell response by accelerating cell cycle progression and the rate of cycle entry, increasing their contribution to the

ensuing GC. This effect occurs over a wide range of initial B cell receptor affinities and correlates with elevated AKT and S6 phosphorylation. Moreover, the resultant increased proliferation can explain the IL-21-mediated promotion of plasma cell differentiation.

We additionally adjusted to discussion as follows:

Page 9, discussion:
These effects are mediated, at least in part, by IL-21 and T cell help additively promoting two key events in triggering cell division, phosphorylation of AKT and S6.

Was changed to:

These effects correlate with IL-21 and T cell help additively promoting two key events in triggering cell division, phosphorylation of AKT and S6.

Referee #2 (Referee #3 TEJ):

Despite the altered conclusions, this manuscript is cleaner and more consistent and generally addresses my previous concerns. The message is a bit more limited than the previous version, but it does have its significance.

My only comment is that I think the authors should extend the discussion of the similarities and differences from the previous paper looking at IL-21 signalling in B cells (Zotos et al 2010). These previous experiments used a non-competitive system as compared to the competitive transfers used here and they found little effect on total numbers of antigen-specific B cells, but reduced numbers of plasma cells and increased numbers of non-GC B cells. Some explanation for the reader of why the current manuscript differs from these previous studies would help to align this new paper with the literature.

We thank the reviewer for highlighting this important point. We extended the discussion as follows:

Discussion, page 11 now additionally includes:

We had previously found that while the proportion of antigen-specific B cells undergoing cell division was significantly reduced in *Il21r*$^{-/-}$ mice from day 5 post immunization (Zotos et al., 2021), their absolute numbers were comparable to WT mice until day 7 (Zotos et al., 2010; Zotos et al., 2021). These earlier studies investigated an endogenous response to a T cell dependent antigen with a high B-cell precursor frequency (Weisel et al, 2016) and the ability to recruit B cells over several days. As a result, rare, antigen-specific B cell numbers early during the response are likely reflecting both recruitment and rate of expansion and the former may compensate for the latter if antigen and T cell help are readily available. In contrast, the adoptive transfer system used in this study effectively has a single wave of WT and *Il21r*$^{-/-}$ SW_{HEL} B cells that are in direct competition within the same immune response and have known, identical starting frequencies and antigen affinities. These factors, in combination with increased in experimental resolution, likely explain the earlier and more pronounced effect of IL-21 on antigen specific B cell numbers reported here.

Dr. Isaak Quast
Monash University
Immunology and Pathology
Burnet tower, 89 Commercial Rd,
Melbourne, Victoria 3004
Australia

Dear Dr. Quast,

I am very pleased to accept your manuscript for publication in the next available issue of EMBO reports. Thank you for your contribution to our journal.

At the end of this email I include important information about how to proceed. Please ensure that you take the time to read the information and complete and return the necessary forms to allow us to publish your manuscript as quickly as possible.

As part of the EMBO publication's Transparent Editorial Process, EMBO reports publishes online a Review Process File to accompany accepted manuscripts. As you are aware, this File will be published in conjunction with your paper and will include the referee reports, your point-by-point response and all pertinent correspondence relating to the manuscript.

If you do NOT want this File to be published, please inform the editorial office within 2 days, if you have not done so already, otherwise the File will be published by default [contact: emboreports@embo.org]. If you do opt out, the Review Process File link will point to the following statement: "No Review Process File is available with this article, as the authors have chosen not to make the review process public in this case."

Thank you again for your contribution to EMBO reports and congratulations on a successful publication. Please consider us again in the future for your most exciting work.

Yours sincerely,

Achim Breiling
Senior Editor
EMBO Reports

****

THINGS TO DO NOW:

Please note that you will be contacted by Wiley Author Services to complete licensing and payment information. The required 'Page Charges Authorization Form' is available here: https://www.embopress.org/pb-assets/embo-site/er_apc.pdf - please download and complete the form and return to embopressproduction@wiley.com

You will receive proofs by e-mail approximately 2-3 weeks after all relevant files have been sent to our Production Office; you should return your corrections within 2 days of receiving the proofs.

Please inform us if there is likely to be any difficulty in reaching you at the above address at that time. Failure to meet our deadlines may result in a delay of publication, or publication without your corrections.

All further communications concerning your paper should quote reference number EMBOR-2022-54677V3 and be addressed to emboreports@wiley.com.

Should you be planning a Press Release on your article, please get in contact with emboreports@wiley.com as early as possible, in order to coordinate publication and release dates.

EMBO Press Author Checklist

Corresponding Author Name: Isaak Quast	
Journal Submitted to: EMBO Reports	
Manuscript Number: # EMBOR-2022-54677	

USEFUL LINKS FOR COMPLETING THIS FORM
The EMBO Journal - Author Guidelines
EMBO Reports - Author Guidelines
Molecular Systems Biology - Author Guidelines
EMBO Molecular Medicine - Author Guidelines

Reporting Checklist for Life Science Articles (updated January 2022)
This checklist is adapted from Materials Design Analysis Reporting (MDAR) Checklist for Authors. MDAR establishes a minimum set of requirements in transparent reporting in the life sciences (see Statement of Task: 10.31222/osf.io/9sm4x). Please follow the journal's guidelines in preparing your manuscript.
Please note that a copy of this checklist will be published alongside your article.

Abridged guidelines for figures
1. Data
The data shown in figures should satisfy the following conditions:
- the data were obtained and processed according to the field's best practice and are presented to reflect the results of the experiments in an accurate and unbiased manner.
- ideally, figure panels should include only measurements that are directly comparable to each other and obtained with the same assay.
- plots include clearly labeled error bars for independent experiments and sample sizes. Unless justified, error bars should not be shown for technical replicates.
- if n<5, the individual data points from each experiment should be plotted. Any statistical test employed should be justified.
- Source Data should be included to report the data underlying figures according to the guidelines set out in the authorship guidelines on Data Presentation.

2. Captions
Each figure caption should contain the following information, for each panel where they are relevant:
- a specification of the experimental system investigated (eg cell line, species name).
- the assay(s) and method(s) used to carry out the reported observations and measurements.
- an explicit mention of the biological and chemical entity(ies) that are being measured.
- an explicit mention of the biological and chemical entity(ies) that are altered/varied/perturbed in a controlled manner.
- the exact sample size (n) for each experimental group/condition, given as a number, not a range;
- a description of the sample collection allowing the reader to understand whether the samples represent technical or biological replicates (including how many animals, litters, cultures, etc.).
- a statement of how many times the experiment shown was independently replicated in the laboratory.
- definitions of statistical methods and measures:
  - common tests, such as t-test (please specify whether paired vs. unpaired), simple χ2 tests, Wilcoxon and Mann-Whitney tests, can be unambiguously identified by name only, but more complex techniques should be described in the methods section;
  - are tests one-sided or two-sided?
  - are there adjustments for multiple comparisons?
  - exact statistical test results, e.g., P values = x but not P values < x;
  - definition of 'center values' as median or average;
  - definition of error bars as s.d. or s.e.m.

Please complete ALL of the questions below.
Select "Not Applicable" only when the requested information is not relevant for your study.

Materials

Newly Created Materials	Information included in the manuscript?	In which section is the information available? (Reagents and Tools Table, Materials and Methods, Figures, Data Availability Section)
New materials and reagents need to be available; do any restrictions apply?	Yes	Materials and Methods

Antibodies	Information included in the manuscript?	In which section is the information available? (Reagents and Tools Table, Materials and Methods, Figures, Data Availability Section)
For **antibodies** provide the following information:<br>- Commercial antibodies: RRID (if possible) or supplier name, catalogue number and or/clone number<br>- Non-commercial: RRID or citation	Yes	Materials and Methods

DNA and RNA sequences	Information included in the manuscript?	In which section is the information available? (Reagents and Tools Table, Materials and Methods, Figures, Data Availability Section)
**Short novel DNA or RNA including primers, probes**: provide the sequences.	Not Applicable	

Cell materials	Information included in the manuscript?	In which section is the information available? (Reagents and Tools Table, Materials and Methods, Figures, Data Availability Section)
**Cell lines:** Provide species information, strain. Provide accession number in repository **OR** supplier name, catalog number, clone number, and/**OR** RRID.	Not Applicable	
**Primary cultures:** Provide species, strain, sex of origin, genetic modification status.	Yes	Materials and Methods
Report if the cell lines were recently **authenticated** (e.g., by STR profiling) and tested for mycoplasma contamination.	Not Applicable	

Experimental animals	Information included in the manuscript?	In which section is the information available? (Reagents and Tools Table, Materials and Methods, Figures, Data Availability Section)
**Laboratory animals or Model organisms:** Provide species, strain, sex, age, genetic modification status. Provide accession number in repository **OR** supplier name, catalog number, clone number, **OR** RRID.	Yes	Materials and Methods
**Animal observed in or captured from the field:** Provide species, sex, and age where possible.	Not Applicable	
Please detail **housing and husbandry conditions**.	Yes	Materials and Methods

Plants and microbes	Information included in the manuscript?	In which section is the information available? (Reagents and Tools Table, Materials and Methods, Figures, Data Availability Section)
**Plants:** provide species and strain, ecotype and cultivar where relevant, unique accession number if available, and source (including location for collected wild specimens).	Not Applicable	
**Microbes:** provide species and strain, unique accession number if available, and source.	Not Applicable	

Human research participants	Information included in the manuscript?	In which section is the information available? (Reagents and Tools Table, Materials and Methods, Figures, Data Availability Section)
If collected and within the bounds of privacy constraints report on age, sex and gender or ethnicity for all study participants.	Not Applicable	

Core facilities	Information included in the manuscript?	In which section is the information available? (Reagents and Tools Table, Materials and Methods, Figures, Data Availability Section)
If your work benefited from core facilities, was their service mentioned in the acknowledgments section?	Yes	Acknowledgements

Design

Study protocol	Information included in the manuscript?	In which section is the information available? (Reagents and Tools Table, Materials and Methods, Figures, Data Availability Section)
If study protocol has been **pre-registered, provide DOI in the manuscript**. For clinical trials, provide the trial registration number **OR** cite DOI.	Not Applicable	
Report the **clinical trial registration number** (at ClinicalTrials.gov or equivalent), where applicable.	Not Applicable	

Laboratory protocol	Information included in the manuscript?	In which section is the information available? (Reagents and Tools Table, Materials and Methods, Figures, Data Availability Section)
Provide DOI OR other citation details if **external detailed step-by-step protocols** are available.	Not Applicable	

Experimental study design and statistics	Information included in the manuscript?	In which section is the information available? (Reagents and Tools Table, Materials and Methods, Figures, Data Availability Section)
Include a statement about **sample size** estimate even if no statistical methods were used.	Yes	Figure Legends
Were any steps taken to minimize the effects of subjective bias when allocating animals/samples to treatment (e.g. **randomization procedure**)? If yes, have they been described?	Yes	Materials and Methods
Include a statement about **blinding** even if no blinding was done.	Yes	Materials and Methods
Describe **inclusion/exclusion criteria** if samples or animals were excluded from the analysis. Were the criteria pre-established?<br><br>If sample or data points were omitted from analysis, report if this was due to attrition or intentional exclusion and provide justification.	Yes	Materials and Methods
For every figure, are **statistical tests** justified as appropriate? Do the data meet the assumptions of the tests (e.g., normal distribution)? Describe any methods used to assess it. Is there an estimate of variation within each group of data? Is the variance similar between the groups that are being statistically compared?	Yes	Figure Legends

Sample definition and in-laboratory replication	Information included in the manuscript?	In which section is the information available? (Reagents and Tools Table, Materials and Methods, Figures, Data Availability Section)
In the figure legends: state number of times the experiment was **replicated** in laboratory.	Yes	Figures Legends
In the figure legends: define whether data describe **technical or biological replicates**.	Yes	Figure Legends

Ethics

Ethics	Information included in the manuscript?	In which section is the information available? (Reagents and Tools Table, Materials and Methods, Figures, Data Availability Section)
Studies involving **human participants**: State details of **authority granting ethics approval** (IRB or equivalent committee(s), provide reference number for approval.	Not Applicable	
Studies involving **human participants**: Include a statement confirming that **informed consent** was obtained from all subjects and that the experiments conformed to the principles set out in the WMA Declaration of Helsinki and the Department of Health and Human Services Belmont Report.	Not Applicable	
Studies involving **human participants:** For publication of **patient photos**, include a statement confirming that consent to publish was obtained.	Not Applicable	
Studies involving experimental **animals**: State details of **authority granting ethics approval** (IRB or equivalent committee(s), provide reference number for approval. Include a statement of compliance with ethical regulations.	Yes	Materials and Methods
Studies involving **specimen and field samples:** State if relevant **permits** obtained, provide details of authority approving study; if none were required, explain why.	Not Applicable	

Dual Use Research of Concern (DURC)	Information included in the manuscript?	In which section is the information available? (Reagents and Tools Table, Materials and Methods, Figures, Data Availability Section)
Could your study fall under dual use research restrictions? Please check biosecurity documents and list of **select agents and toxins** (CDC): https://www.selectagents.gov/sat/list.htm	Not Applicable	
If you used a select agent, is the security level of the lab appropriate and reported in the manuscript?	Not Applicable	
If a study is subject to dual use research of concern regulations, is the name of the **authority granting approval and reference number** for the regulatory approval provided in the manuscript?	Not Applicable	

Reporting

The MDAR framework recommends adoption of discipline-specific guidelines, established and endorsed through community initiatives. Journals have their own policy about requiring specific guidelines and recommendations to complement MDAR.

Adherence to community standards	Information included in the manuscript?	In which section is the information available? (Reagents and Tools Table, Materials and Methods, Figures, Data Availability Section)
State if relevant guidelines or checklists (e.g., **ICMJE, MIBBI, ARRIVE, PRISMA**) have been followed or provided.	Not Applicable	
For **tumor marker prognostic studies,** we recommend that you follow the **REMARK** reporting guidelines (see link list at top right). See author guidelines, under 'Reporting Guidelines'. Please confirm you have followed these guidelines.	Not Applicable	
For **phase II and III randomized controlled trials**, please refer to the **CONSORT** flow diagram (see link list at top right) and submit the CONSORT checklist (see link list at top right) with your submission. See author guidelines, under 'Reporting Guidelines'. Please confirm you have submitted this list.	Not Applicable	

Data Availability

Data availability	Information included in the manuscript?	In which section is the information available? (Reagents and Tools Table, Materials and Methods, Figures, Data Availability Section)
Have **primary datasets** been deposited according to the journal's guidelines (see 'Data Deposition' section) and the respective accession numbers provided in the Data Availability Section?	Not Applicable	
Were **human clinical and genomic datasets** deposited in a public access-controlled repository in accordance to ethical obligations to the patients and to the applicable consent agreement?	Not Applicable	
Are **computational models** that are central and integral to a study available without restrictions in a machine-readable form? Were the relevant accession numbers or links provided?	Not Applicable	
If publicly available data were reused, provide the respective **data citations in the reference list**.	Not Applicable	
